# Testing Serum Albumins and Cyclodextrins as Potential Binders of the Mycotoxin Metabolites Alternariol-3-Sulfate, Alternariol-9-Monomethylether and Alternariol-9-Monomethylether-3-Sulfate

**DOI:** 10.3390/ijms232214353

**Published:** 2022-11-18

**Authors:** Beáta Lemli, Péter Vilmányi, Eszter Fliszár-Nyúl, Balázs Zoltán Zsidó, Csaba Hetényi, Lajos Szente, Miklós Poór

**Affiliations:** 1 Department of Pharmacology, Faculty of Pharmacy, University of Pécs, Rókus u. 2, H-7624 Pécs, Hungary; 2 Green Chemistry Research Group, János Szentágothai Research Centre, University of Pécs, Ifjúság útja 20, H-7624 Pécs, Hungary; 3 Food Biotechnology Research Group, János Szentágothai Research Centre, University of Pécs, Ifjúság útja 20, H-7624 Pécs, Hungary; 4 Pharmacoinformatics Unit, Department of Pharmacology and Pharmacotherapy, Medical School, University of Pécs, Szigeti út 12, H-7624 Pécs, Hungary; 5 CycloLab Cyclodextrin Research & Development Laboratory, Ltd., Illatos út 7, H-1097 Budapest, Hungary

**Keywords:** alternariol sulfates, alternariol monomethyl ether, serum albumin, cyclodextrin, mycotoxin binders

## Abstract

*Alternaria* mycotoxins, including alternariol (AOH), alternariol-9-monomethylether (AME), and their masked/modified derivatives (e.g., sulfates or glycosides), are common food contaminants. Their acute toxicity is relatively low, while chronic exposure can lead to the development of adverse health effects. Masked/modified metabolites can probably release the more toxic parent mycotoxin due to their enzymatic hydrolysis in the intestines. Previously, we demonstrated the complex formation of AOH with serum albumins and cyclodextrins; these interactions were successfully applied for the extraction of AOH from aqueous matrices (including beverages). Therefore, in this study, the interactions of AME, alternariol-3-sulfate (AS), and alternariol-9-monomethylether-3-sulfate (AMS) were investigated with albumins (human, bovine, porcine, and rat) and with cyclodextrins (sulfobutylether-β-cyclodextrin, sugammadex, and cyclodextrin bead polymers). Our major results/conclusions are the following: (1) The stability of mycotoxin–albumin complexes showed only minor species dependent variations. (2) AS and AMS formed highly stable complexes with albumins in a wide pH range, while AME–albumin interactions preferred alkaline conditions. (3) AME formed more stable complexes with the cyclodextrins examined than AS and AMS. (4) Beta-cyclodextrin bead polymer proved to be highly suitable for the extraction of AME, AS, and AMS from aqueous solution. (5) Albumins and cyclodextrins are promising binders of the mycotoxins tested.

## 1. Introduction

**Figure 1:**
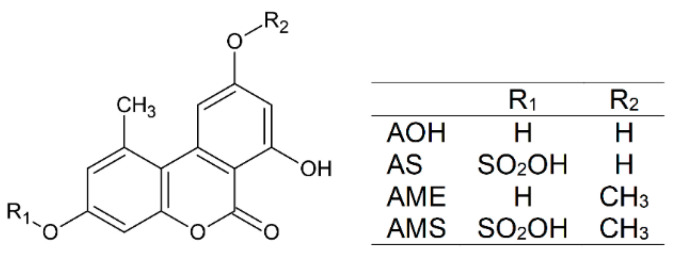
Chemical structures of alternariol (AOH), alternariol-3-sulfate (AS), alternariol-9-monomethylether (AME), and alternariol-9-monomethylether-3-sulfate (AMS).

Mycotoxins are toxic secondary metabolites of filamentous fungi. *Alternaria* species produce several mycotoxins, including alternariol (AOH; Figure 1), alternariol-9-monomethylether (AME; Figure 1), altenuene, altertoxin-I, tenuazonic acid, and tentoxin [1]. *Alternaria* toxins are common food contaminants; they appear in sunflower seeds and oil, grains and grain-based products, tomatoes, apples, fruit and vegetable juices, and alcoholic beverages (e.g., wine and beer) [1,2]. Their acute toxicity is relatively low; however, chronic exposure to *Alternaria* mycotoxins may lead to the development of genotoxic, fetotoxic, and/or endocrine disruptor effects [2,3]. Besides the parent mycotoxins, masked/modified derivatives are also contained in certain food samples, resulting from the metabolism of mycotoxins by plants or by the fungi [4]. These masked/modified metabolites (e.g., sulfates or glycosides) can probably release the parent mycotoxin due to enzymatic hydrolysis in the intestinal tract of mammals [2]. The presence of alternariol-3-sulfate (AS; Figure 1) and alternariol-9-monomethylether-3-sulfate (AMS; Figure 1) have been described in several food samples; they typically occur in tomato products [5,6,7]. Furthermore, AS and AMS are also produced from AOH and AME by sulfotransferases in humans and animals [2,8,9].

Albumin is the most common plasma protein [10,11]. Besides its antioxidant and buffer functions, albumin maintains the oncotic pressure of the blood and carries several endogenous and exogenous compounds in circulation [11]. Structurally, human serum albumin (HSA) is a flexible single polypeptide chain [10,11]. This heart-shaped globular protein is built up from 585 amino acids with a molecular weight of 66.5 kDa. HSA is composed of three homologous domains (I-III) and each domain is built up from two subdomains (A and B) [11,12]. The flexibility and the domain structure of HSA provide several binding pockets for endogenous and exogenous ligands [11]. The conventional bindings sites of drugs and other xenobiotics on HSA are located in Sudlow’s Site I (in subdomain IIA) or in Sudlow’s Site II (in subdomain IIIA) [13,14]. In addition, the Heme site (or FA1; in subdomain IB) has also been reported as a third important drug binding site on the protein [15]. Besides the potential toxicological importance of mycotoxin–albumin interactions, the formation of stable ligand–albumin complexes makes possible the application of albumin as an affinity protein, as has been demonstrated with regard to ochratoxin A [16].

Cyclodextrins (CDs) are cyclic oligosaccharides commercially produced from starch by enzymatic cleavage. The most common CDs are alfa-, beta-, and gamma-CDs built up from six, seven, and eight glucose subunits, respectively. The toroid structure of CDs allows them to host a wide variety of molecules inside their hydrophobic cavity, while their hydrophilic outer surface gives them good aqueous solubility [17]. Sulfobutylether-β-CD (SBECD) is a highly water-soluble and relatively non-toxic CD derivative which is commonly employed by the pharmaceutical industry, due to its negligible nephrotoxic and hemolytic effects [18]. Sugammadex (SGD) is a sulphanylpropanoic acid-substituted γ-CD derivative; it is applied as a medication to rapidly reverse rocuronium- or vecuronium-induced skeletal muscle relaxation [19]. Both SBECD and SGD can even be applied parenterally with good in vivo tolerability [20,21]. Insoluble, but water-swellable, CD polymers show promising results in regard to their application as binders of certain xenobiotics, which can be utilized in analytical sample extraction and/or in the removal of toxic compounds from aqueous solutions [22,23,24]. In addition, β-CD polymers proved to be suitable for the extraction of some mycotoxins (e.g., AOH, ochratoxin A, and zearalenone) from aqueous matrices, including beverages [25,26,27,28]. Importantly, after the toxin extraction, CD polymers can be regenerated with alcohol-water mixtures (due to the displacement of the guest molecules from the CD cavities), and the polymer can be applied again in further extraction cycles [29].

Our recent studies demonstrated the strong interaction of AOH with serum albumins and with certain CDs (including SBECD and SGD) [25,30]. In addition, albumin and β-CD bead polymer (BBP) were successfully applied for the extraction of AOH from beverages, which can be used for analytical sample preparation and/or for the removal of the mycotoxin from aqueous matrices [26]. In the current study, we aimed to investigate the interactions of AS, AME, and AMS with serum albumins (human, bovine, porcine, and rat) and CDs (SBECD, SGD, and CD bead polymers). Our results demonstrate that albumins and BBP are promising binders of AS, AME, and AMS. Since antibody-based products are not available, our data give a good starting point to design albumin- or CD-based strategies for the extraction of these mycotoxins.

## 2. Results and Discussion

### 2.1. Interaction of AS, AME, and AMS with Serum Albumins

**Figure 2:**
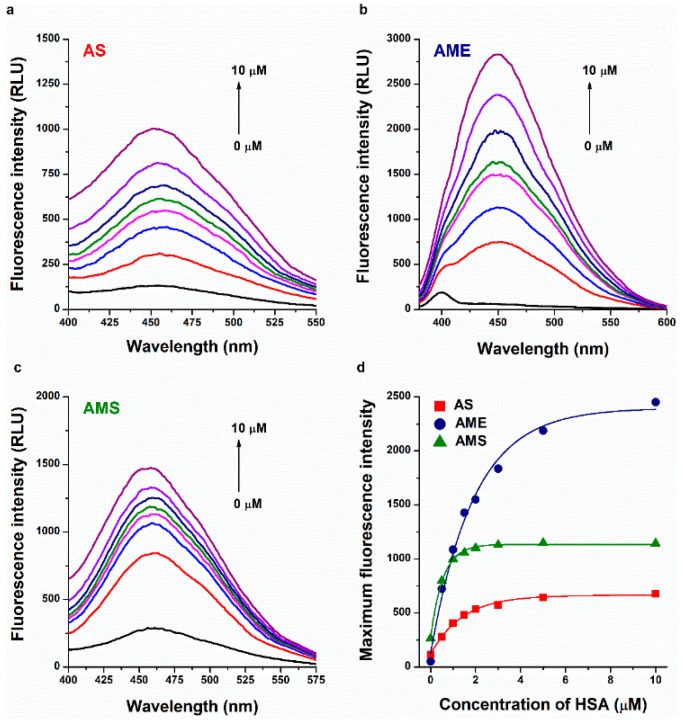
Representative fluorescence emission spectra of AS (**a**), AME (**b**), and AMS (**c**) (each 1.0 μM) in the presence of increasing concentrations (0.0, 0.5, 1.0, 1.5, 2.0, 3.0, 5.0, and 10.0 μM) of HSA in PBS (pH 7.4; λ_ex_ = 335 nm for AS and AMS, and 350 nm for AME). HSA-induced increase in the emission signals of AS (λ_em_ = 455 nm), AME (λ_em_ = 450 nm), and AMS (λ_em_ = 455 nm) (**d**).

Fluorescence spectroscopic studies have been successfully employed to examine the molecular interactions of AOH with serum albumins, CDs, ions, and detergents [25,30,31]. AS, AME, and AMS also exhibit intrinsic fluorescence. Therefore, to test the formation of mycotoxin–albumin complexes, we examined the fluorescence emission spectra of AS, AME, and AMS in the presence of increasing amounts of HSA in PBS (pH 7.4). Even after background correction, HSA induced a strong, gradual increase in the emission signal of each mycotoxin tested (Figure 2), suggesting the formation of ligand–albumin complexes. The water molecules in the hydration shell can partly quench the fluorescence signal of an aromatic fluorophore [32]. The interaction of AS, AME, and AMS with serum albumin results in the partial decomposition of their hydration shells, leading to the decreased quenching effects of water molecules and their increased emission signals [30]. Using these data, we tried to determine the binding constants of mycotoxin–HSA complexes using Hyperquad2006 software (Protonic Software GmbH, Hanau, Germany) [33]; however, we did not get reliable data from this evaluation. Nevertheless, the curves of AS and AMS went to saturation earlier as compared to AME (Figure 2d), suggesting that AS and AMS form more stable complexes with the protein than AME.

**Figure 3:**
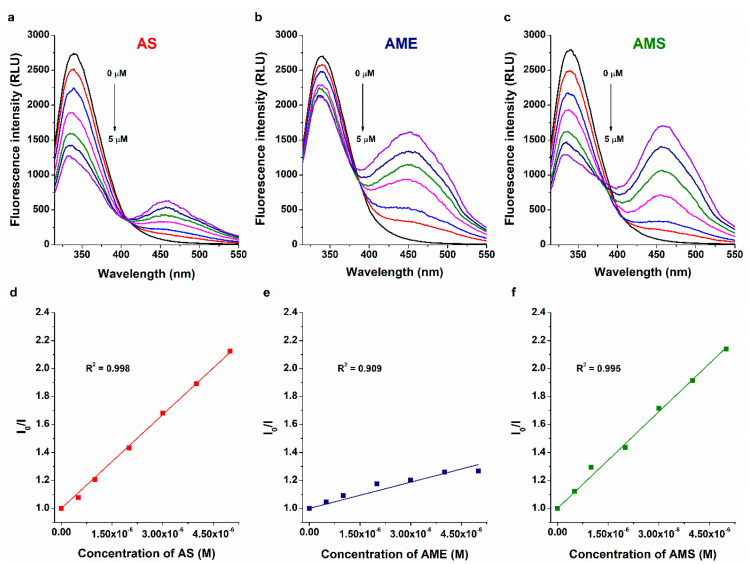
Representative fluorescence emission spectra of HSA (2 μM) in the presence of increasing concentrations (0.0, 0.5, 1.0, 2.0, 3.0, 4.0, and 5.0 μM) of AS (**a**), AME (**b**), and AMS (**c**) in PBS (pH 7.4, λ_ex_ = 295 nm). Stern–Volmer plots of AS–HSA (**d**), AME–HSA (**e**), and AMS–HSA (**f**) interactions (λ_ex_ = 295 nm, λ_em_ = 340 nm).

To determine the binding constants of mycotoxin–HSA complexes, fluorescence quenching and ultracentrifugation studies were performed. The sole tryptophane molecule of HSA (Trp-214) is mainly responsible for the emission signal of the protein [34]. The fluorescence of Trp-214 is sensitive to microenvironmental changes; therefore, the formation of ligand–HSA complexes typically decreases the emission signal of the protein at 340 nm [35]. In quenching experiments, increasing mycotoxin concentrations were added to a standard amount of HSA in PBS (pH 7.4). The first peak at 340 nm belongs to the protein, while the second peaks at higher wavelengths are exerted by the mycotoxins tested (Figure 3). In a concentration-dependent fashion, AS, AME, and AMS induced a gradual decrease in the emission signal of HSA at 340 nm. After correction of the inner-filter effects of mycotoxins [30], data were evaluated using the graphical application of the Stern–Volmer equation (linear fitting; Equation (1)) and by employing Hyperquad software (non-linear fitting) [30,33]. Stern–Volmer plots of AS and AMS showed good linearity, while a lower R^2^ value (0.91) was observed with respect to AME (Figure 3). We hypothesized that this may be a result of the overlapping emission spectra of HSA and AME; however, even after the deconvolution of the two spectra, we did not see relevant changes in the emission data at 340 nm. Based on the Stern–Volmer plot, the log*K_SV_* values of the mycotoxin–HSA complexes were 5.44 ± 0.07 (AS), 4.86 ± 0.05 (AME), and 5.42 ± 0.06 (AMS). In agreement with these results, the evaluations with the Hyperquad software suggested 5.61 ± 0.11, 4.94 ± 0.07, and 5.58 ± 0.08 log*K* values for the AS–HSA, AME–HSA, and AMS–HSA complexes, respectively. Both evaluations suggested 1:1 stoichiometry of complex formation with respect to each mycotoxin–HSA complex examined.

To confirm the results of the spectroscopic studies, ultracentrifugation experiments were also performed, where the free unbound fractions of these mycotoxins were determined in the presence of increasing concentrations of HSA. With ultracentrifugation, albumin and albumin-bound ligands were sedimented, after which the unbound fraction of the ligand molecule was quantified from the protein-free supernatant. Based on these experiments, we calculated 5.50 ± 0.27, 5.34 ± 0.33, and 5.49 ± 0.24 as the log*K* values of the AS–HSA, AME–HSA, and AMS–HSA complexes, respectively (Equation (3)). These data show good correlation with the binding constants determined in the fluorescence quenching studies.

Considering the above-listed results, sulfate metabolites (AS and AMS) form similarly stable complexes with HSA than AOH (log*K* = 5.6) [30]. The binding constant of AME–HSA is lower compared to AOH–HSA, AS–HSA, and AMS–HSA complexes. Nevertheless, the log*K* value of AME–HSA is close to 5, suggesting its high-affinity interaction with the protein. Since AS, AME, and AMS appear in circulation [2,8,9], the formation of highly stable complexes with the protein may affect their toxicokinetics.

**Figure 4:**
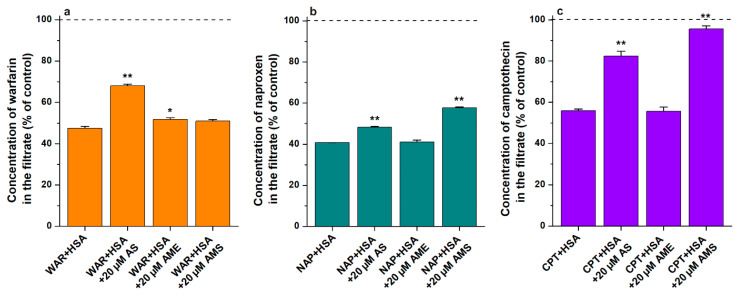
Effects of AS, AME, and AMS on the filtered fractions of warfarin (WAR, Site I; (**a**)), naproxen (NAP, Site II; (**b**)) and *S*-camptothecin (CPT, Heme site; (**c**)). Before filtration, samples contained warfarin and HSA (1.0 and 5.0 μM, respectively), naproxen and HSA (1.0 and 1.5 μM, respectively), or camptothecin and HSA (1.0 and 2.0 μM, respectively) in the absence and presence of AS, AME, or AMS (each 20 μM) in PBS (pH 7.4; * *p* < 0.05, ** *p* < 0.01). Concentrations of site markers in the filtrates were determined by HPLC as has been previously reported [30,36]. Data were compared to the filtered concentration of the corresponding site markers in the absence of albumin (100%; dashed line).

In order to get insight into the binding sites and displacing abilities of AS, AME, and AMS, ultrafiltration studies were performed, employing Site I (warfarin), Site II (naproxen), and Heme site (*S*-camptothecin) markers. HSA and albumin-bound molecules cannot pass through the filter unit with a 30 kDa (or lower) molecular weight cut-off (MWCO) value; therefore, the displacement (or the decreased binding affinity) of a site marker leads to its elevated concentration in the filtrate. AME induced no or only slight effects; it caused a statistically significant (*p* < 0.05) impact only on the filtered fraction of warfarin (Figure 4). AS produced marked increases in the filtered concentrations of warfarin and camptothecin, and its lower but significant (*p* < 0.01) effect was also observed on Site II. AMS did not affect the filtered fraction of warfarin; however, it induced moderate and large elevations of naproxen and camptothecin levels in the filtrates, respectively (Figure 4). Interestingly, in our previous study, AOH strongly displaced warfarin, slightly displaced naproxen, and did not affect the filtered fraction of camptothecin [30]. Since AS and AMS showed complex modulation of the ligand–albumin interactions examined, modeling studies were also performed.

**Figure 5:**
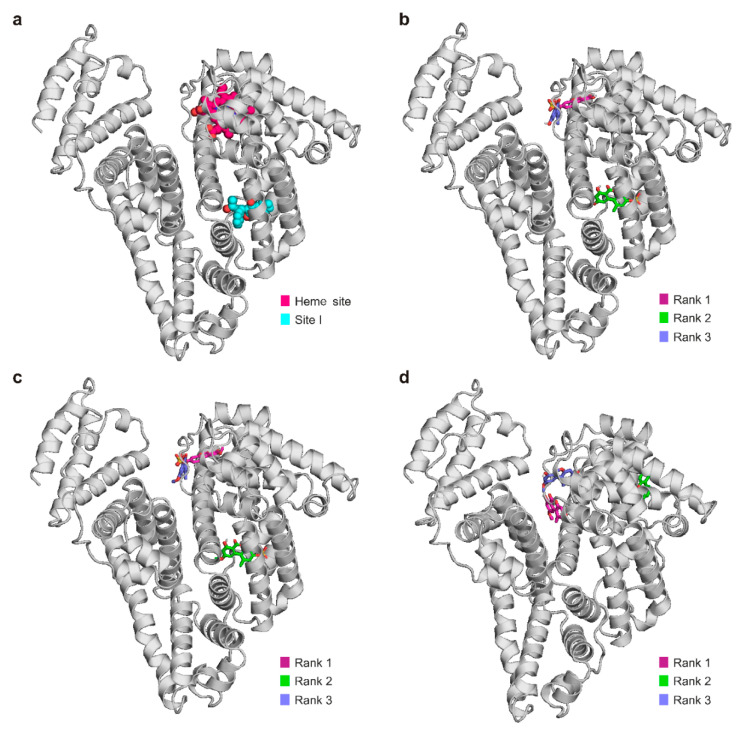
Potential binding sites of AS, AME, and AMS on human serum albumin based on blind docking studies. The protein is represented with grey cartoons, Sudlow’s Site I is highlighted by *S*-warfarin (PDB ID: 1ha2 [37]) with teal spheres, and the Heme site (FA1) is marked by heme (PDB ID: 1n5u [38]) with purple spheres (**a**). We demonstrated the first three ranked binding modes of AS (**b**), AME (**c**), and AMS (**d**) on human serum albumin, where the mycotoxin metabolites were represented with purple (1st rank), green (2nd rank), and blue (3rd rank).

Each compound had its top-ranked binding mode in the Heme site, with AS and AME in the 1st rank and AMS in the 3rd rank (Figure 5). The calculated free energies of AS–HSA and AMS–HSA complexes (in regard to the Heme site) were very similar (−6.4 kcal/mol), while AME showed weaker interaction (−5.2 kcal/mol). These data are in agreement with the binding constants determined (based on fluorescence quenching and ultracentrifugation studies), and also explain the low displacing ability of AME vs. the Heme site marker tested.

AS and AME had 2nd-ranked binding positions in Site I, whereas AMS did not show such a binding mode. In agreement with this latter finding, AMS did not affect the albumin binding of warfarin (Figure 5a). Therefore, it is reasonable to hypothesize that the high-affinity binding site of AMS is located in or close to the Heme site. With respect to Site I, the calculated free energy was significantly weaker for AME (−5.1 kcal/mol) compared to AS (−6.2 kcal/mol), which is in accordance with the slight displacing ability of AME vs. warfarin (Figure 5a). AS has top-ranked binding modes both in Site I and in the Heme site, and it significantly increased the filtered fractions of warfarin and camptothecin in ultrafiltration experiments (Figure 5a,c). Importantly, Site I and the Heme site are allosterically coupled [11]; therefore, their ligands may modulate the interactions of each other via allosteric mechanisms. Spectroscopic studies suggested a 1:1 stoichiometry of complex formation; thus, the high-affinity binding sites of AS and AME can be located in Site I or in the Heme site, but we have no clear evidence. Our previous studies with AOH suggested Site I as the high-affinity binding site of this mycotoxin on HSA [30].

Both AS and AMS increased the filtered fraction of naproxen (Figure 5b). Based on modeling studies, it is unlikely that these modified mycotoxins occupy Site II as their high-affinity binding site. Therefore, the decreased albumin binding of naproxen may a result of its allosteric interaction with sulfate derivatives.

In our previous study, we observed strong species differences in AOH–albumin interactions, where AOH bound to rat albumin with almost eight-fold higher affinity compared to human or porcine albumins [30]. To test the species dependent variations in the albumin binding of AS, AME, and AMS, their interactions were also tested with bovine (BSA), porcine (PSA), and rat (RSA) serum albumins employing fluorescence quenching studies. Table 1 and Table 2 summarize the log*K_SV_* and log*K* values of mycotoxin–albumin complexes, respectively. We did not find large differences; mycotoxins typically formed similarly stable complexes with HSA, BSA, and PSA. RSA bound each compound with the highest affinity; however, the binding constants of mycotoxin–RSA complexes were only approximately three-fold higher compared to the corresponding mycotoxin–HSA complexes (Table 2). These observations demonstrate that each albumin listed can be considered as affinity protein with respect to binding AS, AME, and AMS.

**Figure 6:**
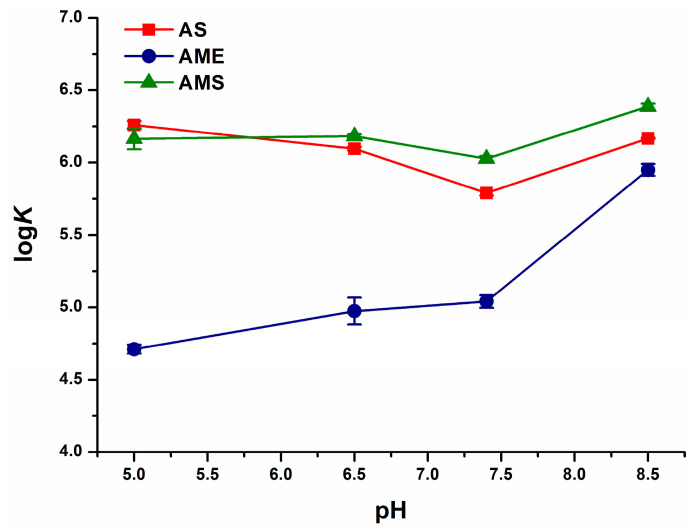
Effects of environmental pH on the binding constants of AS–BSA, AME–BSA, and AMS–BSA complexes (0.05 M sodium acetate, pH 5.0; 0.05 M sodium phosphate, pH 6.5; PBS, pH 7.4; and 0.05 M sodium borate, pH 8.5).

Since BSA is a relatively cheap protein and widely available, we tested the impacts of pH on the binding constants of mycotoxin–BSA complexes. Depending on the environmental pH, albumin appears in its different forms: below pH 2.7 the extended (E) form, between pH 2.7 and 4.3 the fast-migrating (F) form (with increased viscosity, low solubility, and the loss of α-helix), from pH 4.3 to pH 8.0 the normal (N) form (with the heart-shaped structure), and above pH 8.0 the basic (B) form (with the loss of α-helix and an increased affinity towards certain ligands) [11]. We examined the complex formation of mycotoxins with BSA at pH 5.0, pH 6.5, pH 7.4, and pH 8.5. At the pH range tested, the log*K* values of AS–BSA and AMS–BSA complexes showed only minor changes. However, at pH 8.5, AME bound to the protein with approximately ten-fold higher affinity than under acidic and physiological conditions (Figure 6). These observations highlight that the binding ability of the B-form of albumin is considerably better compared to the N-form with respect to AME. Thus, under alkaline circumstances, AME forms similarly stable complexes with BSA than AS and AMS. The binding constants of mycotoxin–HSA complexes tested (Table 2) were typically in the 10^5^ L/mol to 10^6^ L/mol range, showing the formation of high-affinity ligand–albumin complexes and suggesting that albumins are suitable affinity proteins to extract these mycotoxins, as has been demonstrated in regard to AOH [26].

### 2.2. Interaction of AS, AME, and AMS with Cyclodextrins

**Figure 7:**
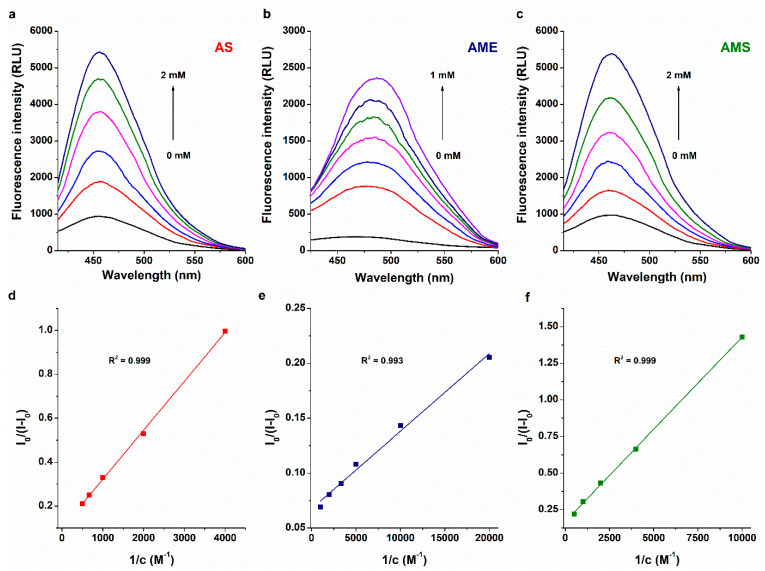
Representative fluorescence spectra of AS (**a**), AME (**b**), and AMS (**c**) (each 1 μM) in the presence of increasing concentrations (0.00, 0.25, 0.50, 1.00, 2.00, 5.00, and 10.0 μM) of sugammadex (SGD) in 0.05 M sodium acetate buffer (pH 5.0; λ_ex_ = 335 nm for AS and AMS, and 350 nm for AME). Benesi–Hildebrand plots of AS–SGD (**d**), AME–SGD (**e**), and AMS–SGD (**f**) complexes (λ_em_ = 455 nm for AS, 485 nm for AME, and 460 nm for AMS).

Since AOH formed stable complexes with SBECD and SGD [39], the interactions of AS, AME, and AMS were also tested with these chemically modified CDs. Similar to the mycotoxin–albumin interactions (Figure 2), the inclusion of AS, AME, and AMS in the CD cavity induce the partial decomposition of the hydration shell of these aromatic fluorophores, leading to a large elevation in their emission signal. Therefore, increasing amounts of CDs were added to standard concentration of mycotoxins in sodium acetate buffer (pH 5.0), then fluorescence emission spectra were recorded. SBECD and SGD induced concentration-dependent elevations in the emission signals of each mycotoxin examined (Figure 7). Based on these data, binding constants of mycotoxin–CD complexes were determined by applying the graphical application of the Benesi–Hildebrand equation (linear fitting; Equation (2)) and employing Hyperquad software (non-linear fitting) [25,33]. Benesi–Hildebrand plots showed excellent fitting with the 1:1 stoichiometry model (Figure 7), and the evaluation with Hyperquad software also suggested the formation of 1:1 mycotoxin–CD complexes. The log*K* values determined with the two evaluation methods showed good correlations (Table 3). AS and AMS formed similarly stable complexes with SBECD and SGD (log*K* ≈ 3); however, compared to the CD complexes of the sulfate metabolites tested, the higher stability of AME–SBECD (three-fold) and AME–SGD (ten-fold) complexes was observed (Table 3). These data demonstrate that similar to AOH [39], AME also produces highly stable complexes with SGD, while the sulfate conjugation of these mycotoxins strongly decreases their binding affinity.

To test the impacts of environmental pH on the complex formation of AS, AME, and AMS with SGD, these interactions were also examined in sodium borate buffer (pH 10.0). Under alkaline circumstances, the binding constants of these mycotoxin–SGD complexes became only slightly (approximately 1.5-fold) higher (log*K*_AS–SGD_ = 3.18 ± 0.58; log*K*_AME–SGD_ = 4.25 ± 0.06; log*K*_AMS–SGD_ = 3.39 ± 0.10) than at pH 5.0 (Table 3). Interestingly, these observations highlight that SGD forms similarly stable complexes with AS, AME, and AMS under acidic and alkaline conditions. In contrast, the interactions of AOH with different CDs proved to be weaker at pH 10.0 (except when cationic CDs were applied), likely due to the ionization of the mycotoxin under alkaline conditions [25]. Considering our recently reported results that certain CDs were able to strongly alleviate the AOH-induced toxicity in cell experiments and/or in zebrafish embryos [39], SBECD and/or SGD seem to be promising binders of AME and the sulfate derivatives tested here.

**Figure 8:**
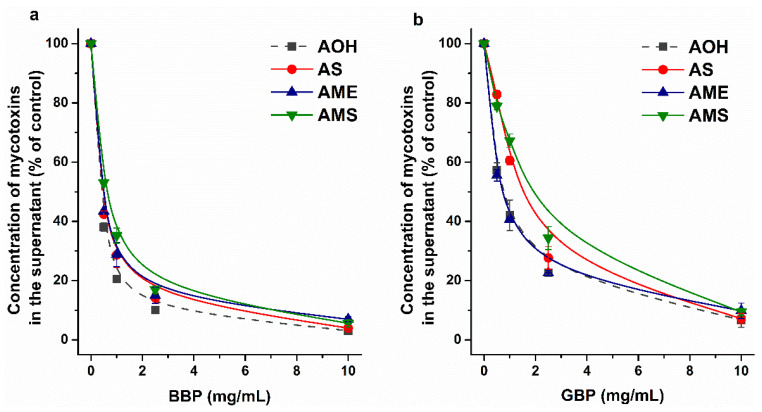
Extraction of AOH, AS, AME, and AMS (each 5 μM) from sodium acetate buffer (0.05 M, pH 5.0) by β-CD bead polymer (BBP; (**a**)) and by γ-CD bead polymer (GBP; (**b**)).

AOH was successfully extracted from aqueous matrices (including beverages) with β-CD bead polymer (BBP) [25,26]. Therefore, the removal of AOH, AS, AME, and AMS by BBP and by γ-CD bead polymer (GBP) were also examined. Since the pH of the beverages typically contaminated with *Alternaria* mycotoxins is acidic [40,41], we performed these investigations in sodium acetate buffer (pH 5.0). BBP extracted similarly high amounts of AOH, AS, AME, and AMS (Figure 8a), showing that the polymer is also a suitable binder of AME and the sulfate derivatives. The highest BBP concentration applied (10 mg/mL) removed approximately 95% of these mycotoxins from the buffer. Herein, GBP was tested for the first time to extract mycotoxins from aqueous solution; however, it proved to be a less effective binder of the mycotoxins examined, compared to BBP (Figure 8). The 0.5–2.5 mg/mL amounts of GBP caused somewhat lower decreases in the concentrations of sulfate metabolites compared to AOH and AME. Nevertheless, GBP (10 mg/mL) was able to extract approximately 90% of AOH, AS, AME, and AMS. The above-listed data clearly demonstrate that CD technology is suitable for the extraction of AS, AME, and AMS from aqueous matrices.

## 3. Materials and Methods

### 3.1. Reagents

Alternariol (AOH) was obtained from Cfm Oskar Tropitzsch GmbH (Marktredwitz, Germany). Alternariol-3-sulfate ammonium salt (AS), alternariol-9-monomethylether (AME), and alternariol-9-monomethylether-3-sulfate ammonium salt (AMS) were purchased from ASCA GmbH (Berlin, Germany). Human serum albumin (HSA), bovine serum albumin (BSA), porcine serum albumin (PSA), rat serum albumin (RSA), racemic warfarin (WAR), racemic naproxen (NAP), and *S*-camptothecin (CPT) were from Merck (Darmstadt, Germany). Sulfobutylether-β-cyclodextrin (SBECD), sugammadex (SGD), insoluble (but water-swellable) β-cyclodextrin bead polymer (BBP; epichlorohydrin cross-linked bead polymer; BCD content: 50 m/m%), and insoluble (but water-swellable) γ-cyclodextrin bead polymer (GBP; epichlorohydrin cross-linked bead polymer; GCD content: 60 m/m%) were obtained from CycloLab Cyclodextrin Research and Development Laboratory, Ltd. (Budapest, Hungary). Acetonitrile (HPLC grade) and dimethyl sulfoxide (DMSO, spectroscopic grade) were from VWR (Debrecen, Hungary) and Fluka (Bucharest, Romania), respectively. Other chemicals used were analytical grade.

Stock solutions of AOH (5 mM) and its metabolites (AS, AME, and AMS; each 10 mM) were prepared in DMSO and stored at −20 °C. Phosphate-buffered saline (PBS; pH 7.4; I = 0.327 M) contained NaCl (137 mmol/L), KCl (2.7 mmol/L), Na_2_HPO_4_ (10 mmol/L), and KH_2_PO_4_ (1.8 mmol/L). In certain experiments 0.05 M sodium acetate (pH 5.0; I = 0. 0.034 M), 0.05 M sodium phosphate (pH 6.5; I = 0.116 M), 0.05 M sodium borate (pH 8.5; I = 0.010 M), and 0.05 M sodium borate (pH 10.0; I = 0.045 M) buffers were applied.

### 3.2. Spectroscopic Studies

Fluorescence emission spectra were collected employing a Hitachi F-4500 fluorescence spectrophotometer (Tokyo, Japan) at 25 °C and in the presence of air. UV–Vis absorption spectra of AS, AME, and AMS were recorded using a Jasco V730 UV–Vis spectrophotometer (Tokyo, Japan). In fluorescence spectroscopic studies, the inner-filter effects of mycotoxins were corrected as has been previously reported [30,42].

To investigate the interaction of mycotoxins with HSA, increasing amounts (final concentrations: 0–10 μM) of the protein were added to AS, AME, or AMS (each 1 μM). After background correction, the HSA-induced changes in the fluorescence emission spectra of mycotoxins were examined (λ_ex_ = 335 nm for AS/AMS and 350 nm for AME; λ_em_ = 455 nm for AS/AMS and 450 nm for AME).

Then, fluorescence quenching studies were also performed, where increasing amounts of AS, AME, or AMS (final concentrations: 0–5 μM) were added to 2 μM concentrations of albumins (HSA, BSA, PSA, or RSA), after which emission spectra were recorded (λ_ex_ = 295 nm). After the correction of the inner-filter effects, mycotoxin-induced changes were evaluated at 340 nm.

Quenching experiments were evaluated by employing the graphical application of the Stern–Volmer equation (linear fitting) and using HyperQuad2006 software (non-linear fitting). Stern–Volmer quenching constants (*K_SV_*; unit: L/mol) were determined based on the Stern–Volmer equation [30]:

(1) $$\frac{I_{0}}{I}=1+K_{SV}\times [Q]$$

where *I*_0_ and *I* are the fluorescence emission intensities of albumin in the absence and presence of the quencher (mycotoxins), respectively; [*Q*] is the molar (mol/L) concentration of mycotoxins. Binding constants (*K*; unit: L/mol) were calculated using HyperQuad2006 software, as has been previously reported [25,30,33].

To examine the interaction of mycotoxins with the two CDs selected (SBECD and SGD), increasing amounts of the CDs (final concentrations: 0–10 mM) were added to mycotoxins (each 1 μM), then fluorescence emission spectra were recorded (λ_ex_ = 335 nm for AS/AMS and 350 nm for AME; λ_em_ = 455 nm for AS, 485 nm for AME, and 460 nm for AMS). Binding constants (*K*, unit: L/mol) of mycotoxin–CD complexes were calculated employing the graphical application of the Benesi–Hildebrand equation (linear fitting) and using Hyperquad software (non-linear fitting). The Benesi–Hildebrand equation [25,43] has been described as:

(2) $$\frac{F_{0}}{\left(F-F_{0}\right)}=\frac{1}{A}+\frac{1}{A\times K\times { [CD]}^{n}}$$

where, *F*_0_ and *F* are the fluorescence emission intensities of the mycotoxin in the absence and presence of CDs, respectively. [*CD*] is the molar concentration (mol/L) of the CD, while *A* is a constant, and *n* is the number of binding sites. Hyperquad evaluations were performed as has been previously reported [25,33].

### 3.3. Ultracentrifugation Studies

To confirm the results of the quenching studies, the unbound fractions of AS, AME, and AMS were quantified in the presence of HSA. Applying the optimal conditions of ultracentrifugation, we can sediment albumin and albumin-bound molecules without the disruption of ligand–HSA interactions [36,44], then the free unbound fraction of the ligand molecule can be quantified from the protein-free supernatant. Samples contained AS, AME, or AMG (each 10 μM) with HSA (20, 60, and 180 μM) in PBS (pH 7.4). These samples (900 μL) were centrifuged for 16 h at 170,000 g and 20 °C in 11 × 34 mm PC tubes (Beckman Coulter, Brea, CA, US), employing a Beckman Coulter Optima MAX-XP tabletop ultracentrifuge (with MLA-130 fixed-angle rotor). Then the concentrations of AS, AME, and AMS were directly analyzed from the supernatants with HPLC-FLD (see details in Section 3.7).

Assuming the 1:1 stoichiometry of complex formation, the binding constants (*K*) were calculated based on the following equation [36]:

(3) $$K=\frac{ [MA]}{ [M]\times [A]}$$

where [*M*], [*A*], and [*MA*] are the molar concentrations (mol/L) of the free unbound mycotoxin, the free unbound albumin, and the mycotoxin–albumin complex, respectively.

### 3.4. Ultrafiltration Studies

To test the binding sites of mycotoxin metabolites on HSA, ultrafiltration experiments were performed, where warfarin, naproxen, and *S*-camptothecin were used as Site I, Site II, and Heme site markers, respectively. Ultrafiltration studies were performed as described earlier [30,36], with minor modifications. Briefly, warfarin and naproxen samples were filtered using Amicon Ultra centrifugal filters (Merck, Darmstadt, Germany) with a 30 kDa molecular weight cut-off (MWCO) value. Camptothecin barely passed through these filters; therefore, the displacement of this Heme site marker was examined employing Pall Microsep Advance centrifugal devices (MWCO: 10 kDa; VWR, Budapest, Hungary). Each filter unit was washed once with water and once with PBS (pH 7.4). Thereafter, samples were centrifuged with a fixed angle rotor for 10 min at 7500 g and 25 °C. Before ultrafiltration, samples contained warfarin (1.0 μM) + HSA (5.0 μM), naproxen (1.0 μM) + HSA (1.5 μM), or camptothecin (1.0 μM) + HSA (2.0 μM) in the absence and presence of AS, AME, or AMS (each 20 μM) in PBS (pH 7.4). The filters used retain HSA (66.5 kDa) and albumin-bound ligands. Therefore, the elevated concentration of the site marker in the filtrate indicates its displacement from the protein [30,36]. The concentrations of warfarin and camptothecin in the filtrate were analyzed by HPLC-FLD, while naproxen was quantified by HPLC-UV as has been previously reported [30,36].

### 3.5. Modeling Studies

The structures of alternariol-3-sulfate (AS), alternariol-9-methyl-ether (AME), and alternariol-9-methyl-ether-3-sulfate (AMS) were built in Maestro (Schrödinger, Maestro Schrödinger Release 2020-4). A subsequent steepest descent and conjugate gradient energy minimization of the ligands were performed with OpenBabel [45]. The resultant structures were further subject to MOPAC [46] geometry optimization with a PM7 parametrization [47], with a gradient norm of 0.001. Gasteiger–Marsili [48] partial charges were assigned to the ligand atoms in AutoDock Tools [49]. Flexibility was allowed on the ligands at all active torsions. These prepared structures were used for docking.

Atomic coordinates of human serum albumin (HSA) were obtained from the Protein Data Bank (PDB) with PDB code 1ao6 [12], according to a previous study [50]. The target molecule was equipped with polar hydrogen atoms and Gasteiger–Marsili partial charges in AutoDock Tools.

Ligands were docked to HSA using AutoDock 4.2.6 [49]. The number of grid points was set to 100 × 100 × 100 at a 0.803 Å grid spacing. The docking box covered the whole surface of the target molecule, and a blind docking investigation was performed [51,52,53]. Lamarckian genetic algorithm was used for global search. One hundred docking runs were performed, and the resultant ligand conformations were ranked by their free energy of binding values [53]. A lower rank indicates a more favorable calculated free energy of binding. Representative docked ligand conformations were used for subsequent evaluations [54].

### 3.6. Extraction of AS, AME, and AMS by CD Bead Polymers

The extraction of mycotoxins AOH, AS, AME, and AMS was tested in sodium acetate buffer (0.05 M, pH 5.0), employing insoluble (but water-swellable) β-CD (BBP) and γ-CD (GBP) bead polymers. A standard concentration of mycotoxins (5 μM) was incubated with increasing amounts of BBP or GBP (0.0, 0.5, 1.0, 2.5, and 10 mg/mL) in a thermomixer for 40 min (1000 rpm, 25 °C). Thereafter, the bead polymer was sedimented by pulse centrifugation (4000 g, 3 s), and mycotoxins were analyzed with HPLC-FLD (see in Section 3.7).

### 3.7. HPLC Analyses

We applied a Jasco HPLC system (Tokyo, Japan) with a binary pump (PU-4180), an autosampler (AS 4050), and a fluorescence detector (FP-920). Chromatographic data were evaluated employing ChromNAV2 software (Jasco). AOH was quantified as has been previously reported [25]. AS, AME, and AMS were analyzed applying the methods described below. Samples (20 μL) were driven through a Security Guard (C18, 4.0 × 3.0 mm; Phenomenex, Torrance, CA, US) precolumn and a Kinetex XB-C18 (250 × 4.6 mm, 5 μm; Phenomenex) analytical column. The isocratic elution was performed with 1 mL/min flow rate, using acetonitrile and 1 mM phosphoric acid (52:48 v/v%) as the mobile phase. AS and AMS were detected at 455 nm (λ_ex_ = 335 nm), while AME was examined at 404 nm (λ_ex_ = 350 nm).

### 3.8. Statistical Analyses

Figures and tables represent mean ± standard error of the mean (SEM), using values from at least three independent experiments. Statistical significance (*p* < 0.05 and *p* < 0.01) was established using a one-way ANOVA (with Tukey post-hoc) test using IBM SPSS Statistics software (IBM, Armonk, NY, USA).

## 4. Conclusions

In summary, the interactions of AME as well as the modified mycotoxins AS and AMS were examined with albumins (HSA, BSA, PSA, and RSA) and with CDs (SBECD, SGD, BBP, GBP). AS and AMS formed highly stable complexes with serum albumins, similar to AOH. The binding constants of AME–albumin complexes were somewhat lower; however, these can still also be considered strong interactions. The binding sites of the mycotoxins tested are likely in Site I and/or in the Heme site. In addition, AS and AMS caused strong and complex modulation with respect to the albumin binding of the site markers tested. We observed only minor species-dependent variations in the albumin binding of AS, AME, and AMS. AS and AMS formed similarly stable complexes with BSA in a wide pH range, while AME–BSA interaction was approximately ten-fold stronger under alkaline conditions than at acidic or physiological pH. AME formed more stable complexes with SBECD and SGD than did AS and AMS; the binding constant of AME–SGD was outstanding among the mycotoxin–CD complexes tested. BBP proved to be equally effective in the extraction of AOH, AS, AME, and AMS from aqueous solution. GBP also induced a large decrease in mycotoxin content; however, it was less effective than BBP. Considering our above-listed observations, albumins and CDs seem to be promising binders of both the parent (AOH and AME) and modified (AS and AMS) mycotoxins examined. On a molar basis, albumins bind these mycotoxins with higher affinity than the CDs tested. Nevertheless, CD polymers can be easily regenerated, and the polymer can be applied again in further extraction cycles. Therefore, our study gives a good starting point for the development of albumin- and CD-based extraction strategies with respect to both the parent (AOH and AME) and the modified (AS and AMS) mycotoxins.

## Figures and Tables

**Table 1:** Decimal logarithmic values of Stern–Volmer quenching constants (*K_SV_*; L/mol) of mycotoxin–albumin complexes, determined based on fluorescence quenching studies.

	HSA log*K_SV_* (±SEM)	BSA log*K_SV_* (±SEM)	PSA log*K_SV_* (±SEM)	RSA log*K_SV_* (±SEM)
AS	5.44 ± 0.07	5.60 ± 0.01	5.57 ± 0.02	5.85 ± 0.03
AME	4.86 ± 0.05	4.93 ± 0.05	4.73 ± 0.06	5.15 ± 0.06
AMS	5.42 ± 0.06	5.81 ± 0.01	5.60 ± 0.02	5.92 ± 0.03

HSA, human serum albumin; BSA, bovine serum albumin; PSA, porcine serum albumin; RSA, rat serum albumin.

**Table 2:** Decimal logarithmic values of binding constants (*K*; L/mol) of mycotoxin–albumin complexes, determined based on fluorescence quenching studies.

	HSA log*K* (±SEM)	BSA log*K* (±SEM)	PSA log*K* (±SEM)	RSA log*K* (±SEM)
AS	5.61 ± 0.11	5.79 ± 0.02	5.78 ± 0.03	6.08 ± 0.03
AME	4.94 ± 0.07	5.04 ± 0.04	4.83 ± 0.05	5.29 ± 0.07
AMS	5.58 ± 0.08	6.03 ± 0.02	5.78 ± 0.03	6.13 ± 0.05

HSA, human serum albumin; BSA, bovine serum albumin; PSA, porcine serum albumin; RSA, rat serum albumin.

**Table 3:** Decimal logarithmic values of the binding constants (*K*; L/mol) with respect to mycotoxin–CD complexes determined by employing the Benesi–Hildebrand (BH) plot and Hyperquad software.

	SBECD		SGD	
	log*K* (±SEM) BH-Plot	log*K* (±SEM) Hyperquad	log*K* (±SEM) BH-Plot	log*K* (±SEM) Hyperquad
AS	3.02 ± 0.01	3.02 ± 0.03	2.83 ± 0.01	2.99 ± 0.03
AME	3.46 ± 0.01	3.56 ± 0.01	3.95 ± 0.01	4.06 ± 0.01
AMS	2.96 ± 0.01	3.08 ± 0.01	2.95 ± 0.05	3.21 ± 0.04

SBECD, sulfobutlyether-β-cyclodextrin; SGD, sugammadex.

## Data Availability

Data will be made available on request.
